# Supplementary material for: Assessing COVID-19 vaccine hesitancy and barriers to uptake in Sub-Saharan Africa
Source: Commun Med (Lond). 2023 Sep 11;3:121. doi: 10.1038/s43856-023-00330-9 (PMC10495410; doi:10.1038/s43856-023-00330-9)
Supplement: Supplementary file 1 — Supplemental Material [file 43856_2023_330_MOESM1_ESM.pdf]

Supplementary Materials for 'Assessing COVID-19 vaccine hesitancy and barriers to uptake in Sub-Saharan Africa'

Philip Wollburg[1]

Yannick Markhof[1,2]

Shelton Kanyanda[3]

Alberto Zezza[1]

[1]Development Data Group, World Bank. [2]UNU-MERIT, United Nations University. [3]National Statistical Office, Zomba, Malawi

Supplementary Note 1: Summary of survey questions

Question	Respondent group	Answer options	Availability
(1) Vaccine uptake			
Have you been vaccinated for COVID-19?	all who know of start of vaccination campaigns	Yes, No, Not sure	all countries
Where did you get vaccinated?	all vaccinated	One location per jab received	all countries
What are your main reasons for getting vaccinated for COVID-19 [apart from protecting your health]?	all vaccinated	Up to two reasons (3 in Kenya)	all countries
(2) Vaccine acceptance among the unvaccinated			
Are you planning to be vaccinated for COVID-19?	all who know of start of vaccination campaigns	Yes, No, Not sure	all countries
When a vaccine to protect you from COVID-19 is available to you, are you planning to be vaccinated?	all who do not know of start of vaccination campaigns	Yes, No, Not sure	BFA, MWI, NGA, TZN
If an approved vaccine to prevent coronavirus was available right now at no cost, would you agree to be vaccinated?	all who do not know of start of vaccination campaigns	Yes, No, Not sure	KEN
What are the main reasons why you want to get vaccinated for COVID-19 [apart from protecting your health]?	all willing but unvaccinated	Up to two reasons (3 in Kenya)	all countries
Why are you not sure or not planning to get vaccinated?	all hesitant	List of reasons for hesitancy	all countries
(3) Barriers of access			
Do you know if your country has started COVID-19 vaccination?	all	Yes, No	BFA, MWI, NGA, TZN
Do you know if a vaccine for COVID-19 is available in Kenya?	all	Yes, No	KEN
Why have you not received the COVID-19 vaccine yet?	all willing but unvaccinated who know of start of vaccination campaigns	Up to two reasons	MWI, NGA, TZN, UGA
What do you think are the main difficulties that you will/would encounter to get the vaccine?	all willing but unvaccinated	List of barriers	KEN
(4) Information sources and channels			
Who/what (institution/body/entity) are your sources of information regarding COVID-19 vaccines?	all who know of start of vaccination campaigns	List of information sources	BFA, MWI, NGA, UGA
Which source of information do you trust the most for reliable information on COVID-19 vaccines?	all who know of start of vaccination campaigns	Select one among mentioned info sources	BFA, MWI, NGA, UGA
Through what channels do you receive the information from the source you trust the most?	all who know of start of vaccination campaigns	Select one info channel	BFA, MWI, NGA, UGA
(5) Ambassadors of vaccination			
Would you be more likely to receive the COVID-19 vaccine if any of the following individuals/authorities receive or recommend the vaccine?	all hesitant	Select yes or no for list of individuals/authoriti es	all countries
Whose recommendation to get vaccinated for COVID-19 would you trust the most?	all hesitant that had named at least one individual/authority that could encourage them	Select one among previously selected	BFA, MWI, NGA, TZN, UGA

		potential ambassadors	
How likely are you to encourage others to get the COVID-19 vaccine?	all willing to get vaccinated or already vaccinated	Five point Likert scale	BFA, MWI, NGA, TZN, UGA
(6) Social factors in vaccine attitude formation and uptake			
Out of 10 people in your community, how many do you think have been vaccinated or are willing to be vaccinated for COVID-19?	all	Number from 0-10	BFA, MWI, NGA, TZN
Out of 10 people in your community, how many do you think are willing to be vaccinated for COVID-19?	all	Number from 0-10	UGA
Who in your household mainly decides whether the adult household members will get vaccinated for COVID-19?	all who know of start of vaccination campaigns	Each for themselves; All adults together; Household head; Name other household member	BFA, MWI, NGA, TZN

Supplementary Table 1: Estimated vaccine acceptance

	Burkina Faso	Kenya	Malawi	Nigeria	Tanzania	Uganda
Overall	74.4	95.1	75.1	78.4	63.3	90.8
	(71.5 to 77.2)	(93.4 to 96.9)	(71.6 to 78.7)	(76.2 to 80.5)	(60.4 to 66.2)	(88.9 to 92.8)
Unvaccinated	57.3	86.4	59.1	70.5	56.5	34.4
	(53.0 to 61.5)	(81.8 to 91.0)	(53.9 to 64.3)	(67.7 to 73.4)	(53.2 to 59.7)	(25.9 to 42.9)
Men	74.9	95.6	77.6	81.1	65.3	90.7
	(71.8 to 78.0)	(93.5 to 97.7)	(73.1 to 82.0)	(78.5 to 83.7)	(61.8 to 68.7)	(88.0 to 93.5)
Women	72.3	94.7	71.8	71.4	59.1	91.0
	(65.9 to 78.6)	(92.0 to 97.4)	(66.0 to 77.6)	(67.3 to 75.6)	(54.0 to 64.3)	(88.2 to 93.8)
Urban	58.8	95.6	69.9	75.3	59.8	87.2
	(54.6 to 62.9)	(93.5 to 97.7)	(62.8 to 76.9)	(71.3 to 79.3)	(54.7 to 64.8)	(82.8 to 91.7)
Rural	81.8	94.8	76.4	79.7	65.0	92.6
	(78.2 to 85.3)	(92.4 to 97.3)	(72.3 to 80.4)	(77.1 to 82.4)	(61.4 to 68.5)	(90.7 to 94.5)
p-value men vs. women	0.473	0.019	0.116	<0.001	0.054	0.892
p-value urban vs. rural	<0.001	0.020	0.123	0.095	0.109	0.031
N (overall)	1,847	5,633	1,447	2,934	2,196	1,872
N (unvaccinated)	1,157	2,018	863	2,010	1,791	252
N (male)	1,415	2,605	853	2,053	1,523	1,021
N (female)	432	3,019	594	881	670	851
N (urban)	1,141	3,020	547	1,181	809	469
N (rural)	706	2,613	900	1,753	1,387	1,403

Note: The table compares estimated acceptance rates for COVID-19 vaccines. The breakdown for different demographics includes those already vaccinated. All values in percent. 95% confidence intervals in parentheses. P-values are from two-sided t-tests of differences between two groups.

Supplementary Table 2: Reasons for pending vaccination

**Reasons**	Malawi	Nigeria	Tanzania
Currently ineligible	1.9	3.5	1.7
	(-0.0 to 3.8)	(1.8 to 5.1)	(0.5 to 3.0)
Don't know how to get	3.2	39.6	7.8
	(0.4 to 6.0)	(35.3 to 44.0)	(5.1 to 10.5)
Too crowded at vax site	3.8	6.7	0.8
	(1.1 to 6.6)	(4.5 to 8.9)	(0.1 to 1.5)
Facility not disability friendly	0.0	0.8	0.9
		(0.2 to 1.3)	(0.2 to 1.7)
Too far away	13.6	12.0	12.2
	(8.8 to 18.3)	(9.2 to 14.8)	(9.2 to 15.2)
Not enough vaccines	18.6	9.6	9.1
	(13.6 to 23.6)	(6.9 to 12.4)	(6.6 to 11.6)
Unable to register	0.2	2.0	1.4
	(-0.1 to 0.4)	(0.8 to 3.3)	(0.4 to 2.4)
Work commitments	5.3	11.7	28.0
	(2.6 to 8.0)	(8.6 to 14.8)	(24.1 to 32.0)
Domestic commitments	5.0	1.5	7.2
	(1.9 to 8.1)	(0.4 to 2.6)	(4.9 to 9.4)
Religious reasons	0.5	0.2	0.4
	(-0.2 to 1.2)	(0.0 to 0.4)	(0.0 to 0.7)
Medical reasons	8.0	3.6	6.9
	(3.5 to 12.4)	(1.8 to 5.4)	(4.8 to 9.0)
Waiting for appointment	2.0	9.0	3.3
	(0.5 to 3.6)	(6.3 to 11.7)	(1.8 to 4.7)
Afraid of side effects	35.2	5.8	0.0
	(28.4 to 42.0)	(3.7 to 7.8)	
Other reason	8.4	7.8	26.1
	(4.0 to 12.9)	(5.5 to 10.2)	(22.2 to 30.0)
N	493	1,098	992

Note: Reasons for pending vaccination despite being willing to get vaccinated. All values in percent. 95% confidence intervals in parentheses.

Supplementary Table 3: Reasons for pending vaccination by residence

Reasons	Malawi		Nigeria		Tanzania	
	Rural	Urban	Rural	Urban	Rural	Urban
Currently ineligible	1.9	1.7	3.4	3.6	2.0	1.2
	(-0.3 to 4.2)	(-1.6 to 4.9)	(1.8 to 5.1)	(0.0 to 7.5)	(0.3 to 3.7)	(0.2 to 2.3)
Don't know how to get	3.8	0.3	41.9	34.8	8.6	6.1
	(0.5 to 7.2)	(-0.3 to 1.0)	(36.4 to 47.4)	(27.6 to 42.0)	(5.3 to 11.8)	(1.2 to 11.0)
Too crowded at vax site	3.6	5.1	5.7	9.0	0.8	0.8
	(0.4 to 6.8)	(0.4 to 9.8)	(3.0 to 8.3)	(5.1 to 12.9)	(0.0 to 1.7)	(0.0 to 1.8)
Facility not disability friendly	0.0	0.0	0.7	0.8	1.1	0.6
			(0.0 to 1.4)	(0.0 to 1.9)	(0.1 to 2.0)	(0.0 to 1.7)
Too far away	15.1	6.4	13.6	8.4	14.4	7.1
	(9.6 to 20.6)	(-1.4 to 14.2)	(9.9 to 17.3)	(4.6 to 12.2)	(10.6 to 18.3)	(3.0 to 11.1)
Not enough vaccines	20.7	8.4	9.3	10.4	11.6	3.4
	(14.9 to 26.6)	(1.9 to 15.0)	(5.9 to 12.7)	(5.8 to 15.0)	(8.1 to 15.1)	(1.5 to 5.4)
Unable to register	0.2	0.0	2.1	1.8	1.6	0.9
	(-0.1 to 0.5)	(. to .)	(0.4 to 3.9)	(0.5 to 3.2)	(0.2 to 3.0)	(0.0 to 1.8)
Work commitments	3.5	13.5	9.5	16.4	24.0	37.2
	(1.2 to 5.9)	(3.3 to 23.7)	(5.9 to 13.1)	(10.4 to 22.3)	(19.4 to 28.6)	(29.6 to 44.9)
Domestic commitments	4.9	5.9	0.7	3.2	7.1	7.3
	(1.3 to 8.5)	(0.7 to 11.1)	(0.0 to 1.5)	(0.3 to 6.1)	(4.3 to 10.0)	(3.6 to 10.9)
Religious reasons	0.6	0.0	0.0	0.5	0.3	0.4
	(-0.3 to 1.4)	(. to .)	(0.0 to 0.1)	(0.0 to 1.1)	(0.0 to 0.7)	(0.0 to 1.2)
Medical reasons	7.4	10.5	2.8	5.3	5.8	9.4
	(2.6 to 12.3)	(-1.0 to 22.0)	(0.7 to 4.8)	(1.7 to 8.9)	(3.4 to 8.1)	(5.1 to 13.8)
Waiting for appointment	2.0	2.0	10.6	5.6	3.2	3.4
	(0.3 to 3.8)	(-1.0 to 5.0)	(7.0 to 14.2)	(1.8 to 9.4)	(1.3 to 5.1)	(1.1 to 5.7)
Afraid of side effects	32.8	46.3	4.4	8.6	0.0	0.0
	(25.3 to 40.4)	(31.2 to 61.4)	(2.1 to 6.8)	(4.8 to 12.4)		
Other reason	8.2	9.6	8.1	7.2	25.5	27.4
	(3.3 to 13.0)	(-1.7 to 20.9)	(5.0 to 11.2)	(4.1 to 10.3)	(20.8 to 30.3)	(20.5 to 34.4)
N	177	316	653	447	660	332

Note: Reasons for pending vaccination despite being willing to get vaccinated, by urban/rural. All values in percent. 95% confidence intervals in parentheses.

Supplementary Table 4: Reasons for pending vaccination by gender

Reasons	Malawi		Nigeria		Tanzania	
	Female	Male	Female	Male	Female	Male
Currently ineligible	1.6	2.1	3.1	3.6	0.8	2.1
	(-0.4 to 3.6)	(-0.8 to 5.0)	(0.0 to 7.4)	(1.9 to 5.3)	(0.0 to 1.7)	(0.5 to 3.8)
Don't know how to get	2.1	3.9	39.5	39.7	10.1	7.0
	(-0.7 to 4.9)	(-0.2 to 8.0)	(31.0 to 47.9)	(34.6 to 44.8)	(4.1 to 16.1)	(4.0 to 9.9)
Too crowded at vax site	1.4	5.3	8.5	6.2	1.5	0.5
	(-0.4 to 3.2)	(1.0 to 9.6)	(3.5 to 13.5)	(3.8 to 8.6)	(0.0 to 3.6)	(0.0 to 1.0)
Facility not disability friendly	0.0	0.0	1.2	0.6	0.6	1.0
			(0.0 to 2.5)	(0.0 to 1.2)	(0.0 to 1.9)	(0.1 to 1.9)
Too far away	12.4	14.3	8.0	13.2	8.1	13.7
	(4.2 to 20.5)	(8.5 to 20.2)	(3.0 to 13.0)	(9.9 to 16.5)	(3.4 to 12.9)	(10.0 to 17.4)
Not enough vaccines	12.6	22.3	10.6	9.4	6.4	10.1
	(6.2 to 18.9)	(15.3 to 29.3)	(4.5 to 16.7)	(6.3 to 12.4)	(2.2 to 10.7)	(7.1 to 13.2)
Unable to register	0.0	0.3	0.5	2.5	1.2	1.4
		(-0.2 to 0.7)	(0.0 to 1.2)	(0.9 to 4.1)	(0.0 to 2.7)	(0.1 to 2.7)
Work commitments	0.7	8.1	8.1	12.8	20.1	31.0
	(0.0 to 1.4)	(3.8 to 12.4)	(2.4 to 13.8)	(9.1 to 16.4)	(13.5 to 26.7)	(26.2 to 35.8)
Domestic commitments	6.4	4.2	2.2	1.3	12.0	5.4
	(1.8 to 11.0)	(0.0 to 8.4)	(0.0 to 4.4)	(0.0 to 2.6)	(6.1 to 17.9)	(3.3 to 7.5)
Religious reasons	0.0	0.8	0.5	0.1	0.8	0.2
		(-0.4 to 1.9)	(0.0 to 1.2)	(0.0 to 0.2)	(0.0 to 1.7)	(0.0 to 0.5)
Medical reasons	17.0	2.4	9.4	1.8	13.4	4.4
	(7.2 to 26.7)	(-1.0 to 5.8)	(3.2 to 15.7)	(0.5 to 3.1)	(7.4 to 19.4)	(2.7 to 6.1)
Waiting for appointment	1.7	2.3	3.8	10.6	2.4	3.6
	(-0.2 to 3.5)	(0.1 to 4.5)	(1.5 to 6.1)	(7.2 to 14.1)	(0.6 to 4.2)	(1.6 to 5.5)
Afraid of side effects	42.9	30.4	7.0	5.4	0.0	0.0
	(31.4 to 54.4)	(22.0 to 38.7)	(3.4 to 10.6)	(3.0 to 7.8)		
Other reason	9.5	7.7	10.8	6.9	28.0	25.4
	(0.7 to 18.4)	(3.1 to 12.3)	(4.9 to 16.8)	(4.5 to 9.3)	(20.5 to 35.5)	(20.8 to 30.0)
N	333	160	270	830	277	715

Note: Reasons for pending vaccination despite being willing to get vaccinated, by gender. All values in percent. 95% confidence intervals in parentheses.

Supplementary Table 5: Barriers of access (Kenya)

Barriers	Overall	Rural	Urban	Female	Male
Currently ineligible	7.4	6.1	10.0	7.4	7.3
	(3.1 to 11.6)	(0.8 to 11.3)	(2.8 to 17.1)	(2.3 to 12.5)	(0.6 to 14.1)
Too far away	23.5	24.8	20.9	22.1	24.8
	(17.7 to 29.2)	(17.1 to 32.5)	(13.1 to 28.7)	(14.8 to 29.5)	(16.0 to 33.6)
Don't know how to get	2.6	2.6	2.7	1.7	3.5
	(0.1 to 5.1)	(-1.0 to 6.1)	(0.4 to 4.9)	(0.2 to 3.2)	(-1.1 to 8.1)
Too crowded at vax site	27.8	28.0	27.3	30.2	25.4
	(21.6 to 33.9)	(19.5 to 36.5)	(20.2 to 34.4)	(21.7 to 38.7)	(16.6 to 34.2)
Facility not disability friendly	0.1	0.1	0.2	0.1	0.1
	(0.0 to 0.2)	(-0.0 to 0.1)	(-0.0 to 0.3)	(-0.0 to 0.1)	(-0.0 to 0.3)
No transport	5.3	4.9	6.0	8.4	2.3
	(2.3 to 8.3)	(1.4 to 8.5)	(0.4 to 11.5)	(2.7 to 14.1)	(0.6 to 3.9)
Not enough vaccines	25.8	26.6	24.4	25.1	26.5
	(20.0 to 31.6)	(18.5 to 34.6)	(17.8 to 31.0)	(18.0 to 32.2)	(17.4 to 35.6)
Unable to register	0.8	0.9	0.5	1.1	0.5
	(-0.0 to 1.6)	(-0.2 to 2.1)	(0.1 to 0.8)	(-0.5 to 2.7)	(0.2 to 0.8)
Too expensive to obtain	0.5	0.3	1.1	0.2	0.8
	(-0.1 to 1.1)	(0.0 to 0.5)	(-0.7 to 2.9)	(-0.0 to 0.4)	(-0.4 to 2.0)
Discouraged in community	0.4	0.4	0.3	0.3	0.5
	(0.1 to 0.7)	(-0.0 to 0.9)	(0.0 to 0.6)	(-0.1 to 0.7)	(-0.0 to 1.0)
No barriers	37.9	38.7	36.4	34.7	41.0
	(31.4 to 44.5)	(29.9 to 47.6)	(27.8 to 45.0)	(26.7 to 42.7)	(30.9 to 51.2)
Other barrier	0.0	0.0	0.0	0.1	0.0
	(-0.0 to 0.1)	(-0.0 to 0.1)	(-0.0 to 0.1)	(-0.0 to 0.1)	(-0.0 to 0.0)
N	1,732	839	893	952	777

Note: Main anticipated difficulties to get vaccinated. All values in percent. 95% confidence intervals in parentheses.

Supplementary Table 6: Vaccination location

**Location**	Burkina Faso	Kenya	Malawi	Nigeria	Tanzania	Uganda
Hospital	13.7	59.9	43.6	29.5	31.8	34.0
	(10.4 to 17.1)	(55.2 to 64.6)	(37.1 to 50.2)	(24.7 to 34.4)	(25.1 to 38.5)	(30.6 to 37.4)
Clinic	0.4	6.1	11.5	14.7	1.6	0.9
	(0.0 to 0.7)	(2.8 to 9.5)	(8.0 to 15.0)	(10.9 to 18.5)	(0.0 to 3.6)	(0.1 to 1.7)
Health center	45.4	18.3	23.4	30.7	45.1	41.7
	(40.0 to 50.8)	(14.5 to 22.1)	(17.5 to 29.4)	(26.3 to 35.2)	(38.1 to 52.2)	(38.2 to 45.2)
Pharmacy	0.1	0.0	0.0	0.5	0.1	0.1
	(0.0 to 0.3)	(-0.0 to 0.1)		(0.0 to 1.3)	(0.0 to 0.2)	(0.0 to 0.2)
Senior home	0.2	0.0	0.0	1.2	0.0	0.3
	(0.0 to 0.5)	(-0.0 to 0.0)		(0.0 to 2.8)	(0.0 to 0.1)	(0.0 to 0.8)
Mass vaccination site	35.1	8.9	20.0	12.6	11.9	16.5
	(29.9 to 40.3)	(6.7 to 11.1)	(14.3 to 25.7)	(9.2 to 16.0)	(7.6 to 16.2)	(13.9 to 19.0)
At work	1.6		1.7	3.1	1.2	1.2
	(0.5 to 2.8)		(0.2 to 3.1)	(1.5 to 4.8)	(0.2 to 2.1)	(0.4 to 2.0)
Religious center	0.8		0.3	1.8	3.5	2.4
	(0.0 to 1.6)		(0.0 to 0.6)	(0.0 to 3.7)	(0.8 to 6.2)	(1.3 to 3.6)
Other location	3.1	6.7	0.0	5.5	6.3	11.7
	(1.6 to 4.7)	(4.7 to 8.8)		(3.0 to 8.0)	(2.9 to 9.7)	(9.5 to 13.9)
N	690	3,586	584	921	403	1,618

Note: Vaccination location. All values in percent. 95% confidence intervals in parentheses.

Supplementary Table 7: Reasons for vaccination

**Reason**	Burkina Faso	Kenya	Malawi	Nigeria	Tanzania	Uganda
Protecting own health only	60.8	22.7	89.9	64.1	76.8	65.1
	(55.6 to 66.0)	(18.4 to 26.9)	(86.5 to 93.2)	(59.2 to 69.0)	(70.7 to 83.0)	(61.7 to 68.6)
Protecting other's health	26.7	68.6	5.0	9.3	16.0	16.1
	(22.2 to 31.1)	(64.1 to 73.1)	(2.6 to 7.3)	(6.5 to 12.1)	(10.6 to 21.4)	(13.5 to 18.7)
Government mandate	6.9		1.1	18.7	4.0	23.3
	(4.3 to 9.5)		(0.1 to 2.2)	(14.7 to 22.7)	(1.5 to 6.5)	(20.3 to 26.4)
Attend school	0.3	0.4	0.0	0.2	0.0	1.1
	(0.0 to 0.8)	(0.0 to 0.8)	(0.0 to 0.0)	(0.0 to 0.5)		(0.1 to 2.0)
Employer mandate	0.7	12.1	1.4	1.6	0.9	4.9
	(0.1 to 1.2)	(9.5 to 14.6)	(0.4 to 2.4)	(0.2 to 2.9)	(0.0 to 2.0)	(3.2 to 6.6)
Avoid public health measures	4.4		0.9	3.2	0.5	9.6
	(2.0 to 6.7)		(0.0 to 2.0)	(1.0 to 5.4)	(0.0 to 1.2)	(7.6 to 11.7)
Take part in social life	2.1	0.2	1.3	3.1	2.6	3.7
	(0.6 to 3.6)	(0.1 to 0.3)	(0.0 to 2.7)	(1.2 to 5.0)	(0.4 to 4.9)	(2.4 to 5.0)
Be able to travel	10.7		1.3	10.4	3.1	8.3
	(7.6 to 13.7)		(0.0 to 2.6)	(7.6 to 13.2)	(0.4 to 5.8)	(6.4 to 10.2)
People in community did it too	4.4		0.1	2.3	0.1	3.9
	(2.0 to 6.8)		(0.0 to 0.2)	(1.3 to 3.2)	(0.0 to 0.2)	(2.6 to 5.2)
Received material incentive	0.0		0.0	0.0	0.0	0.0
				(0.0 to 0.1)		
Other reason	1.4	29.7	0.2	1.3	4.1	0.9
	(0.0 to 2.9)	(25.8 to 33.7)	(0.0 to 0.4)	(0.4 to 2.2)	(0.8 to 7.5)	(0.3 to 1.5)
N	690	3,586	584	921	403	1,620

Note: Reasons for getting vaccinated. 'Other' mainly comprises of people citing ethical reasons as their motivation in Kenya (29.2%). All values in percent. 95% confidence intervals in parentheses.

Supplementary Table 8: Reasons for hesitancy

**Reason**	Burkina Faso	Kenya	Malawi	Nigeria	Tanzania	Uganda
Do not think vaccine works	33.2	9.5	15.1	4.7	10.5	1.3
	(27.1 to 39.3)	(3.3 to 15.8)	(9.3 to 20.9)	(2.6 to 6.8)	(7.7 to 13.3)	(0.0 to 2.9)
Worried about side effects	36.5	86.0	45.8	20.8	41.3	29.6
	(30.5 to 42.6)	(77.8 to 94.1)	(37.7 to 53.8)	(16.3 to 25.3)	(36.4 to 46.2)	(19.0 to 40.2)
Already had COVID-19	0.3			0.3	0.1	2.5
	(0.0 to 0.8)			(0.0 to 0.6)	(0.0 to 0.3)	(0.0 to 6.3)
Not enough at risk	3.9	6.1	4.8	18.7	2.8	0.6
	(1.6 to 6.2)	(1.4 to 10.8)	(0.9 to 8.6)	(14.3 to 23.0)	(1.1 to 4.6)	(0.0 to 1.5)
Don't trust vaccines generally	4.8	5.0	18.4	11.6	19.5	17.7
	(2.0 to 7.5)	(1.8 to 8.2)	(12.2 to 24.5)	(8.0 to 15.2)	(15.6 to 23.4)	(8.5 to 26.9)
Against my religion	0.4	1.9	4.9	3.1	1.4	0.3
	(0.0 to 1.3)	(-0.2 to 4.1)	(1.3 to 8.5)	(1.5 to 4.7)	(0.5 to 2.3)	(-0.3 to 0.8)
Infection risk at facility	0.3	1.7	0.2	1.7	0.1	1.6
	(0.0 to 0.6)	(0.2 to 3.1)	(-0.2 to 0.5)	(0.3 to 3.1)	(0.0 to 0.3)	(0.0 to 3.5)
Facility too hard to reach	0.0	7.5	0.8	1.5	2.1	2.5
		(-1.8 to 16.8)	(-0.3 to 1.9)	(0.0 to 3.4)	(0.7 to 3.5)	(0.0 to 5.9)
Do not have time	2.2	0.4	2.9	1.9	2.2	1.4
	(0.7 to 3.6)	(-0.3 to 1.0)	(-0.7 to 6.4)	(0.0 to 4.0)	(0.8 to 3.7)	(0.0 to 2.8)
Vax available here not effective	10.3		1.7	3.0	0.4	1.3
	(7.0 to 13.7)		(-0.6 to 3.9)	(1.3 to 4.6)	(0.0 to 0.9)	(0.0 to 3.1)
Unsure if I get the vax I want	2.6		6.9	1.5	2.2	1.8
	(1.0 to 4.2)		(3.1 to 10.6)	(0.5 to 2.5)	(0.8 to 3.6)	(0.0 to 5.4)
Not a priority	8.7		21.6	11.0	10.9	13.4
	(5.5 to 11.8)		(14.4 to 28.8)	(6.8 to 15.3)	(7.9 to 13.9)	(6.5 to 20.2)
COVID-19 does not exist	8.9			16.3	2.6	2.1
	(5.4 to 12.5)			(11.7 to 20.9)	(1.0 to 4.1)	(-2.0 to 6.3)
Medical reasons	0.6		0.2	2.3	2.2	17.9
	(0.0 to 1.2)		(-0.1 to 0.5)	(0.8 to 3.7)	(0.8 to 3.5)	(9.2 to 26.7)
Do not trust the government	6.1		1.8	5.0	1.7	
	(3.3 to 9.0)		(-0.4 to 4.1)	(2.6 to 7.3)	(0.3 to 3.2)	
Other reason	5.5	0.0	1.7	13.7	16.0	6.1
	(2.5 to 8.5)	(-0.0 to 0.1)	(0.2 to 3.2)	(9.7 to 17.6)	(12.1 to 19.8)	(0.7 to 11.5)
Do not think it is safe		45.2				
		(27.2 to 63.2)				
N	526	313	366	609	769	156

Note: Reasons for being hesitant to get vaccinated. All values in percent. 95% confidence intervals in parentheses.

Supplementary Table 9: Most trusted information sources

**Information Source**	Burkina Faso	Malawi	Nigeria	Uganda
Medical professional	45.2	34.3	28.7	30.4
	(41.0 to 49.4)	(30.1 to 38.6)	(25.7 to 31.7)	(27.3 to 33.4)
Scientists and epidemiologists	0.1	0.0	0.3	0.5
	(0.0 to 0.3)		(0.1 to 0.6)	(0.0 to 1.1)
Celebrities and influencers	0.0	0.9	2.3	0.2
	(0.0 to 0.1)	(0.3 to 1.6)	(1.3 to 3.3)	(0.0 to 0.5)
NGO outreach programs	0.4	1.4	1.0	0.8
	(0.1 to 0.7)	(0.3 to 2.6)	(0.4 to 1.6)	(0.1 to 1.4)
Other outreach programs	0.3	2.3	1.0	1.0
	(0.0 to 0.7)	(0.9 to 3.6)	(0.5 to 1.6)	(0.5 to 1.6)
Local government	5.7	0.5	10.1	24.1
	(4.0 to 7.4)	(0.0 to 1.3)	(8.2 to 12.1)	(21.3 to 26.9)
Federal government	0.3	0.0	8.8	0.3
	(0.0 to 0.7)		(7.2 to 10.4)	(0.0 to 0.8)
State government	6.2	0.0	6.4	7.4
	(4.4 to 8.0)		(5.0 to 7.8)	(5.6 to 9.2)
Family and neighbors	5.6	4.1	12.8	4.0
	(3.9 to 7.2)	(2.2 to 6.0)	(10.7 to 14.8)	(2.7 to 5.3)
Religious organizations	0.9	2.3	3.7	2.5
	(0.1 to 1.7)	(0.6 to 4.0)	(2.7 to 4.7)	(1.4 to 3.6)
Traditional healer	0.4	0.0	0.0	0.0
	(0.0 to 0.9)			(0.0 to 0.1)
Traditional ruler	1.8	2.0	1.5	0.5
	(0.7 to 2.9)	(0.9 to 3.1)	(0.7 to 2.4)	(0.0 to 1.0)
Media	26.5	34.4	10.9	27.9
	(22.9 to 30.0)	(30.3 to 38.4)	(9.1 to 12.7)	(24.9 to 30.9)
Other info source	0.4	0.4	2.1	0.3
	(0.0 to 0.7)	(0.0 to 0.9)	(1.1 to 3.2)	(0.0 to 0.6)
None	1.7	4.1	5.1	0.0
	(1.0 to 2.4)	(2.5 to 5.6)	(3.8 to 6.4)	(0.0 to 0.1)
Trust all equally	4.6	13.3	5.2	0.1
	(3.2 to 5.9)	(10.6 to 16.0)	(3.8 to 6.5)	(0.0 to 0.2)
N	1,744	1,443	2,488	1,859

Note: Most trusted information sources. All values in percent. 95% confidence intervals in parentheses.

Supplementary Table 10: Most trusted information sources by vaccine acceptance

Information Source	Burkina Faso		Malawi		Nigeria		Uganda	
	**Hesitant**	**Willing**	**Hesitant**	**Willing**	**Hesitant**	**Willing**	**Hesitant**	**Willing**
Medical professional	33.5	49.2	32.5	35.0	18.7	31.2	33.1	30.1
	(26.9 to 40.1)	(44.3 to 54.1)	(24.2 to 40.8)	(30.2 to 39.7)	(13.7 to 23.8)	(27.9 to 34.5)	(22.1 to 44.2)	(26.9 to 33.3)
Scientists and epidemiologists	0.5	0.0	0.0	0.0	0.0	0.4	0.1	0.6
	(0.0 to 1.0)	(0.0 to 0.1)			(0.0 to 0.1)	(0.1 to 0.7)	(0.0 to 0.4)	(0.0 to 1.2)
Celebrities and influencers	0.0	0.0	0.3	1.1	2.9	2.1	0.0	0.2
		(0.0 to 0.1)	(0.0 to 1.0)	(0.2 to 1.9)	(0.2 to 5.7)	(1.1 to 3.2)		(0.0 to 0.5)
NGO outreach programs	0.2	0.5	0.1	1.9	0.4	1.1	0.0	0.8
	(0.0 to 0.4)	(0.1 to 0.8)	(0.0 to 0.3)	(0.4 to 3.4)	(0.0 to 0.9)	(0.5 to 1.8)		(0.1 to 1.5)
Other outreach programs	0.1	0.4	1.8	2.4	1.5	0.9	0.0	1.1
	(0.0 to 0.4)	(0.0 to 0.8)	(0.0 to 4.3)	(0.8 to 4.0)	(0.0 to 3.0)	(0.4 to 1.5)		(0.5 to 1.7)
Local government	3.0	6.6	0.0	0.7	3.2	11.9	19.3	24.6
	(0.8 to 5.2)	(4.5 to 8.8)	(0.0 to 0.1)	(0.0 to 1.7)	(1.0 to 5.3)	(9.5 to 14.2)	(10.7 to 27.9)	(21.6 to 27.5)
Federal government	1.0	0.0	0.0	0.0	7.1	9.2	0.0	0.3
	(0.0 to 2.8)	(0.0 to 0.0)			(3.2 to 11.0)	(7.4 to 11.0)		(0.0 to 0.8)
State government	8.1	5.6	0.0	0.0	5.7	6.6	11.5	7.0
	(4.4 to 11.7)	(3.4 to 7.7)			(2.6 to 8.8)	(5.0 to 8.2)	(3.9 to 19.1)	(5.2 to 8.8)
Family and neighbors	4.5	5.9	3.5	4.3	17.1	11.7	6.2	3.8
	(1.9 to 7.0)	(3.9 to 7.9)	(1.4 to 5.6)	(2.0 to 6.7)	(12.4 to 21.7)	(9.5 to 13.9)	(2.4 to 10.0)	(2.4 to 5.2)
Religious organizations	0.3	1.1	1.1	2.7	5.4	3.3	1.0	2.7
	(0.0 to 0.6)	(0.1 to 2.2)	(0.0 to 2.6)	(0.7 to 4.7)	(2.3 to 8.4)	(2.3 to 4.3)	(0.0 to 2.0)	(1.5 to 3.8)
Traditional healer	1.0	0.2	0.0	0.0	0.0	0.0	0.0	0.0
	(0.0 to 2.9)	(0.0 to 0.4)						(0.0 to 0.1)
Traditional ruler	0.8	2.1	1.2	2.2	1.4	1.6	0.3	0.5
	(0.0 to 1.9)	(0.7 to 3.6)	(0.0 to 2.3)	(0.8 to 3.6)	(0.0 to 3.2)	(0.6 to 2.5)	(0.0 to 0.8)	(0.0 to 1.1)
Media	34.1	23.9	37.6	33.3	12.7	10.5	28.4	27.8
	(27.2 to 40.9)	(20.1 to 27.7)	(29.8 to 45.4)	(28.7 to 37.8)	(8.4 to 17.0)	(8.6 to 12.4)	(18.0 to 38.9)	(24.7 to 31.0)
Other info source	1.4	0.0	0.4	0.4	4.2	1.6	0.0	0.3
	(0.0 to 2.9)		(0.0 to 1.2)	(0.0 to 1.0)	(1.0 to 7.4)	(0.5 to 2.7)		(0.0 to 0.6)
None	6.5	0.1	11.4	1.6	14.0	2.8	0.0	0.0
	(3.8 to 9.1)	(0.0 to 0.2)	(6.2 to 16.6)	(0.6 to 2.6)	(9.4 to 18.7)	(1.7 to 4.0)		(0.0 to 0.1)
Trust all equally	5.3	4.3	10.1	14.3	5.6	5.1	0.0	0.1
	(3.0 to 7.6)	(2.8 to 5.8)	(6.1 to 14.1)	(10.9 to 17.7)	(2.3 to 8.9)	(3.7 to 6.5)		(0.0 to 0.2)
N	483	1,261	366	1,077	477	2,021	152	1,708

Note: Most trusted information sources by vaccine acceptance. All values in percent. 95% confidence intervals in parentheses.

Supplementary Table 11: Vaccine ambassadors

**Ambassador**	Burkina Faso	Kenya	Malawi	Nigeria	Tanzania	Uganda
At least some ambassador	42.1	44.0	36.1	58.2	77.3	64.4
	(35.8 to 48.3)	(25.2 to 62.9)	(27.2 to 45.0)	(52.3 to 64.1)	(73.1 to 81.4)	(51.8 to 77.0)
Family	28.3	17.6	29.9	42.7	12.1	24.8
	(21.5 to 35.1)	(8.2 to 27.1)	(21.2 to 38.6)	(36.9 to 48.6)	(8.9 to 15.4)	(14.7 to 34.9)
Religious leader	23.4	11.2	25.4	44.0	20.4	23.3
	(17.1 to 29.7)	(3.9 to 18.6)	(17.6 to 33.2)	(38.0 to 50.0)	(16.2 to 24.6)	(13.3 to 33.2)
Medical professional	34.5	34.8	26.6	40.1	31.9	52.7
	(28.1 to 40.9)	(15.3 to 54.3)	(19.1 to 34.2)	(34.5 to 45.7)	(27.2 to 36.5)	(40.0 to 65.4)
Community leader	22.4	7.3	19.3	35.5	9.5	17.0
	(16.1 to 28.7)	(3.2 to 11.4)	(12.4 to 26.2)	(29.9 to 41.1)	(6.4 to 12.6)	(8.2 to 25.7)
Traditional healer	19.2	5.8	11.0	24.4	2.8	2.3
	(13.0 to 25.3)	(2.2 to 9.4)	(5.9 to 16.2)	(19.8 to 29.0)	(1.2 to 4.3)	(0.1 to 4.5)
Scientist and epidemiologists	21.8	23.1	16.2	32.3	13.4	15.9
	(15.5 to 28.1)	(4.0 to 42.1)	(10.1 to 22.3)	(27.7 to 37.0)	(10.1 to 16.8)	(8.2 to 23.7)
Celebrities and influencers	16.9	5.8	13.5	22.6	4.2	9.3
	(10.8 to 23.0)	(2.2 to 9.4)	(8.0 to 19.0)	(18.4 to 26.8)	(2.2 to 6.2)	(2.4 to 16.2)
Other ambassador	2.3	5.9	0.0	1.1	37.2	2.3
	(0.5 to 4.2)	(1.9 to 9.8)	(0.0 to 0.1)	(0.1 to 2.2)	(32.3 to 42.2)	(0.0 to 4.7)
N	526	313	366	609	769	129

Note: People whose recommendation would make the hesitant more likely to get vaccinated. All values in percent. 95% confidence intervals in parentheses.

Supplementary Table 12: Information channels

**Channel**	Burkina Faso	Malawi	Nigeria	Uganda
In person	44.9	61.7	42.8	52.7
	(40.9 to 48.9)	(57.3 to 66.0)	(39.9 to 45.7)	(49.4 to 56.1)
Posters, billboards and flyers	9.5	2.4	3.2	7.3
	(7.3 to 11.6)	(0.8 to 4.1)	(2.0 to 4.3)	(5.6 to 9.0)
Radio	67.0	51.1	58.8	70.6
	(63.1 to 70.9)	(47.0 to 55.3)	(55.7 to 61.9)	(67.7 to 73.5)
TV	36.1	4.5	25.2	25.6
	(32.3 to 39.9)	(2.8 to 6.2)	(22.2 to 28.3)	(22.6 to 28.5)
SMS	2.6	3.0	5.7	2.5
	(1.2 to 4.1)	(1.6 to 4.4)	(4.4 to 7.0)	(1.4 to 3.5)
Phone	5.0	2.8	11.1	12.5
	(3.5 to 6.6)	(1.4 to 4.2)	(9.2 to 12.9)	(10.2 to 14.8)
Newspaper	6.6	0.7	5.6	6.2
	(4.8 to 8.5)	(0.3 to 1.0)	(4.3 to 6.9)	(4.5 to 7.8)
Social media	8.5	2.9	10.3	3.4
	(6.6 to 10.3)	(1.4 to 4.3)	(8.6 to 12.0)	(2.3 to 4.5)
Other internet channel	0.7	0.0	3.7	1.2
	(0.0 to 1.3)	(0.0 to 0.1)	(2.5 to 4.9)	(0.4 to 2.0)
Other information channel	1.3	2.5	3.0	1.1
	(0.4 to 2.3)	(1.2 to 3.7)	(1.8 to 4.2)	(0.5 to 1.7)
N	1,738	1,442	2,493	1,870

Note: Information channels through which the most trusted information on COVID-19 was obtained. All values in percent. 95% confidence intervals in parentheses.

Supplementary Table 13: Perceived vaccine acceptance

**Estimate**	Burkina Faso	Malawi	Nigeria	Tanzania	Uganda
Estimated national acceptance	74.4	75.1	78.4	63.3	90.8
	(71.5 to 77.2)	(71.1 to 79.1)	(75.8 to 80.9)	(60.2 to 66.3)	(88.9 to 92.8)
Average perceived acceptance	43.2	47.7	59.1	43.6	61.1
	(40.6 to 45.8)	(45.1 to 50.3)	(56.8 to 61.3)	(40.6 to 46.7)	(59.6 to 62.6)
Perceived acceptance (hesitant)	22.7	32.5	25.7	25.6	48.1
	(19.2 to 26.2)	(27.8 to 37.2)	(20.9 to 30.5)	(20.8 to 30.3)	(43.4 to 52.8)
Perceived acceptance (willing)	49.3	52.5	64.8	51.5	62.3
	(46.7 to 51.9)	(49.9 to 55.1)	(62.5 to 67.0)	(48.1 to 55.0)	(60.7 to 63.8)
N (national)	1,847	1,447	2,934	2,196	1,872
N (perceived)	1,551	1,287	1,995	1,416	1,811
N (hesitant)	389	315	280	407	134
N (willing)	1,162	972	1,714	1,009	1,677

Note: Average perceived acceptance of COVID-19 vaccines in one's community. All values in percent. 95% confidence intervals in parentheses.

Supplementary Table 14: Decisionmaker within the household

**Decisionmaker**	Burkina Faso	Malawi	Nigeria	Tanzania	Uganda
Each adult for themselves	50.9	42.0	18.3	37.1	42.0
	(46.7 to 55.2)	(37.6 to 46.5)	(15.2 to 21.5)	(34.0 to 40.3)	(38.8 to 45.2)
All adults together	6.6	17.9	6.6	18.2	20.8
	(4.9 to 8.3)	(15.3 to 20.5)	(5.2 to 8.0)	(15.8 to 20.5)	(18.1 to 23.6)
Household head	42.3	39.4	69.0	44.4	36.8
	(37.9 to 46.8)	(35.4 to 43.4)	(65.5 to 72.6)	(41.1 to 47.7)	(33.7 to 40.0)
Other household member	0.1	0.6	6.0	0.3	0.4
	(0.0 to 0.3)	(0.0 to 1.4)	(4.3 to 7.8)	(0.1 to 0.6)	(0.0 to 0.8)
N	1,744	1,343	2,386	2,131	1,870

Note: Decisionmaker within the household about vaccine uptake of adult household members. All values in percent. 95% confidence intervals in parentheses.

Supplementary Table 15: Likelihood to encourage others

**Likelihood**	Burkina Faso	Malawi	Nigeria	Tanzania	Uganda
1. Very likely	22.9	80.7	72.1	76.4	59.3
	(18.2 to 27.7)	(75.0 to 86.4)	(67.3 to 76.9)	(70.7 to 82.2)	(55.8 to 62.9)
2. Somewhat likely	58.4	13.0	23.6	18.4	29.8
	(52.9 to 63.8)	(8.2 to 17.8)	(19.0 to 28.3)	(13.2 to 23.5)	(26.4 to 33.1)
3. Neither likely nor unlikely	12.1	3.8	1.8	2.5	6.0
	(8.5 to 15.8)	(1.2 to 6.4)	(0.6 to 3.1)	(0.0 to 5.0)	(4.3 to 7.8)
4. Somewhat unlikely	6.5	0.3	1.4	1.4	3.1
	(3.6 to 9.4)	(0.0 to 0.6)	(0.2 to 2.5)	(0.0 to 3.1)	(1.9 to 4.2)
5. Very unlikely	0.1	2.2	1.1	1.3	1.8
	(0.0 to 0.2)	(0.3 to 4.0)	(0.3 to 1.8)	(0.0 to 3.4)	(0.9 to 2.7)
N	690	584	921	402	1,618

Note: Likelihood to encourage others to get vaccinated among those already vaccinated. All values in percent. 95% confidence intervals in parentheses.

Supplementary Table 16: General Adult Population vs. Phone Survey Sample

Characteristic	Burkina Faso		Kenya		Malawi		Nigeria		Tanzania		Uganda	
	General Adult	Phone Survey	General Adult	Phone Survey	General Adult	Phone Survey	General Adult	Phone Survey	General Adult	Phone Survey	General Adult	Phone Survey
Age	35.5	46.3	36.0	35.3	34.3	41.7	35.9	46.0	34.8	46.6	34.4	44.5
Female	54.2	20.8	51.2	50.8	51.3	42.2	51.5	28.0	53.1	32.7	51.8	46.0
Education: None	77.1	79.9	-	-	67.6	51.3	35.5	31.9	-	-	46.5	44.4
Education: Primary	13.7	9.4	-	-	14.6	33.5	17.8	18.6	-	-	37.5	32.1
Education: Secondary	8.2	8.4	-	-	15.3	12.2	41.6	41.3	-	-	7.3	16.9
Education: Tertiary	1.0	2.3	-	-	2.5	2.9	5.1	8.2	-	-	8.7	6.6
N	24,396	1,846	52,841	5,633	8,588	1,446	15,230	2,937	8,989	2,193	8,763	1,867

Note: Comparison of individual characteristics between general adult population (from nationally representative, face-to-face pre-COVID-19 survey) and phone survey respondents. Data for education not available in Kenya and Tanzania.
