# Supplementary material for: Assessing COVID-19 vaccine hesitancy and barriers to uptake in Sub-Saharan Africa
Source: Commun Med (Lond). 2023 Sep 11;3:121. doi: 10.1038/s43856-023-00330-9 (PMC10495410; doi:10.1038/s43856-023-00330-9)
Supplement: Supplementary file 3 — Reporting Summary [file 43856_2023_330_MOESM3_ESM.pdf]

nature portfolio

Corresponding author(s): Philip Wollburg
Last updated by author(s): May 8, 2023

Reporting Summary

Nature Portfolio wishes to improve the reproducibility of the work that we publish. This form provides structure for consistency and transparency in reporting. For further information on Nature Portfolio policies, see our Editorial Policies and the Editorial Policy Checklist.

Statistics

For all statistical analyses, confirm that the following items are present in the figure legend, table legend, main text, or Methods section.

n/a	Confirmed	
☐	☒	The exact sample size (*n*) for each experimental group/condition, given as a discrete number and unit of measurement
☐	☒	A statement on whether measurements were taken from distinct samples or whether the same sample was measured repeatedly
☐	☒	The statistical test(s) used AND whether they are one- or two-sided<br>*Only common tests should be described solely by name; describe more complex techniques in the Methods section.*
☐	☒	A description of all covariates tested
☒	☐	A description of any assumptions or corrections, such as tests of normality and adjustment for multiple comparisons
☐	☒	A full description of the statistical parameters including central tendency (e.g. means) or other basic estimates (e.g. regression coefficient) AND variation (e.g. standard deviation) or associated estimates of uncertainty (e.g. confidence intervals)
☒	☐	For null hypothesis testing, the test statistic (e.g. *F*, *t*, *r*) with confidence intervals, effect sizes, degrees of freedom and *P* value noted<br>*Give P values as exact values whenever suitable.*
☒	☐	For Bayesian analysis, information on the choice of priors and Markov chain Monte Carlo settings
☒	☐	For hierarchical and complex designs, identification of the appropriate level for tests and full reporting of outcomes
☒	☐	Estimates of effect sizes (e.g. Cohen's *d*, Pearson's *r*), indicating how they were calculated

Our web collection on statistics for biologists contains articles on many of the points above.

Software and code

Policy information about availability of computer code

Data collection	The survey instrument used in this study was coded using the World Bank's free software Survey Solutions. Subsequently, Survey Solutions' computer-assisted telephone interviewing (CATI) capabilities were used for data collection.
Data analysis	Authors used Stata/MP 17.0 for data analysis. Full replication code is available on the Harvard Dataverse at https://doi.org/10.7910/DVN/ONQAHA

For manuscripts utilizing custom algorithms or software that are central to the research but not yet described in published literature, software must be made available to editors and reviewers. We strongly encourage code deposition in a community repository (e.g. GitHub). See the Nature Portfolio guidelines for submitting code & software for further information.

Data

Policy information about availability of data

All manuscripts must include a data availability statement. This statement should provide the following information, where applicable:

- Accession codes, unique identifiers, or web links for publicly available datasets
- A description of any restrictions on data availability
- For clinical datasets or third party data, please ensure that the statement adheres to our policy

The data and code required to replicate the tables and figures used in this study have been made publicly available on the Harvard Dataverse at https://doi.org/10.7910/DVN/ONQAHA

The raw data and questionnaires are available through the World Bank's microdata library:
• Burkina Faso (https://microdata.worldbank.org/index.php/catalog/3768)
• Kenya (https://microdata.worldbank.org/index.php/catalog/3774 - data available upon request with country team)
• Malawi (https://microdata.worldbank.org/index.php/catalog/3766)
• Nigeria (https://microdata.worldbank.org/index.php/catalog/4444)
• Tanzania (https://microdata.worldbank.org/index.php/catalog/4542)
• Uganda (https://microdata.worldbank.org/index.php/catalog/3765)

The data for Figure 1 is also available from the Our World in Data GitHub page at https://covid.ourworldindata.org/data/owid-covid-data.csv
Any remaining data are available from the corresponding author on reasonable request.

Human research participants

Policy information about studies involving human research participants and Sex and Gender in Research.

Reporting on sex and gender	Information on sex was collected based on self-reported information from the main respondent.
Population characteristics	The study population was selected from nationally representative samples of households interviewed in recent in-person surveys. In Kenya, an additional sample of households was drawn via random digit dialing. In each household, one main respondent over the age of 15 was interviewed, who was selected to be knowledgeable of the affairs of the household
Recruitment	The high frequency phone surveys (HFPS) used in this study are re-contact surveys with national scope whose samples are drawn from nationally representative samples of households interviewed in recent in-person surveys. In Kenya, an additional sample of households was drawn via random digit dialing. Phone survey samples may not be fully representative of the general population, because of limited mobile phone coverage, non-response, and attrition, especially in lower-income contexts. To mitigate these concerns, sampling weights were recalibrated using propensity score and post-stratification methods, which were shown to mitigate sample selection biases in the HFPS. In each household, one main respondent over the age of 15 was interviewed, who was selected to be knowledgeable of the affairs of the household and its members to provide reliable responses, though this selection overrepresents certain population groups such as men and household heads.<br><br>The pre-COVID, face-to-face national household surveys that served as sampling frames were<br>• Burkina Faso: Enquête Harmonisée sur les Conditions de Vie des Ménages (EHCVM) 2018/19<br>• Kenya: Kenya Integrated Household Budget Survey (KIHBS) 2015/16<br>• Malawi: Integrated Household Panel Survey (IHPS) 2019<br>• Nigeria: General Household Survey-Panel (GHS-Panel) 2018/19<br>• Tanzania: National Panel Survey 2014/15 and Household Budget Survey 2017/18<br>• Uganda: Uganda National Panel Survey 2019/20
Ethics oversight	Each phone survey was implemented by the respective national statistical office (NSO). The NSO conducts the survey as the sole official statistical authority in the country and in accordance with the respective National Statistical Act, which exempts the NSO from institutional ethics approvals. Informed consent was received from all survey respondents in each country. The World Bank does not require institutional ethics approval for household surveys that are partly or fully financed by the World Bank, including the national phone surveys in Burkina Faso, Kenya, Malawi, Nigeria,Tanzania, and Uganda that inform our research.

Note that full information on the approval of the study protocol must also be provided in the manuscript.

Field-specific reporting

Please select the one below that is the best fit for your research. If you are not sure, read the appropriate sections before making your selection.

☐ Life sciences ☒ Behavioural & social sciences ☐ Ecological, evolutionary & environmental sciences

For a reference copy of the document with all sections, see nature.com/documents/nr-reporting-summary-flat.pdf

Behavioural & social sciences study design

All studies must disclose on these points even when the disclosure is negative.

Study description	This study uses quantitative, observational data from a standardized survey conducted in six countries across East and West Africa (Burkina Faso, Kenya, Malawi, Nigeria, Tanzania, and Uganda).
Research sample	The high frequency phone surveys (HFPS) used in this study are re-contact surveys with national scope whose samples are drawn from nationally representative samples of households interviewed in recent in-person surveys. In Kenya, an additional sample of households was drawn via random digit dialing. Phone survey samples may not be fully representative of the general population, because of limited mobile phone coverage, non-response, and attrition, especially in lower-income contexts. To mitigate these

	concerns, sampling weights were recalibrated using propensity score and post-stratification methods, which were shown to mitigate sample selection biases in the HFPS. In each household, one main respondent over the age of 15 was interviewed, who was selected to be knowledgeable of the affairs of the household and its members to provide reliable responses, though this selection overrepresents certain population groups such as men and household heads.
Sampling strategy	To select respondents for the HFPS, the pre-COVID household surveys listed below served as sampling frames. The pre-COVID face-to-face surveys attempted to collect phone numbers from all household members (or alternatively a reference contact such as a neighbor). The sampling for the HFPS started with all enumeration areas covered in the latest pre-COVID household survey. All households with at least one phone number for a household member or reference contact were selected for interviewing. Chapter 2.1 of Brubaker et al. (2021) contains a country-by-country discussion of contact protocols. The target sample size for the HFPS was calculated in order to ensure that, at a minimum, it would be sufficient to detect a 10 percentage point change in the key indicators (COVID-19 knowledge and behavior and labor market impacts) in between rounds with 90% power and 95% confidence at the national level. Notably, sample size requirements for reliable detection of changes across survey rounds are substantially higher than what is required for reliable point estimates within a single round as in the case of our study. A more detailed discussion of the sampling process is provided in Himelein et al. (2020). The sample size resulting from the sampling strategy is summarized in Table 1.<br><br>Pre-COVID face-to-face surveys that served as sampling frames:<br>• Burkina Faso: Enquête Harmonisée sur les Conditions de Vie des Ménages (EHCVM) 2018/19<br>• Kenya: Kenya Integrated Household Budget Survey (KIHBS) 2015/16<br>• Malawi: Integrated Household Panel Survey (IHPS) 2019<br>• Nigeria: General Household Survey-Panel (GHS-Panel) 2018/19<br>• Tanzania: National Panel Survey 2014/15 and Household Budget Survey 2017/18<br>• Uganda: Uganda National Panel Survey 2019/20
Data collection	To ensure smooth implementation and common standards across countries, the launch of the first round of HFPS was proceeded by three days of piloting the questionnaire, CATI technology, survey protocols, and monitoring mechanisms. Questionnaire guidelines and templates were first elaborated by the World Bank's COVID-19 questionnaire working group and served as the backbone of the HFPS implemented in each national statistical office. Interviewers for the survey were selected out of a pool of existing enumerators with experience conducting (LSMS-ISA) household surveys. As such, all enumerators had undergone previous (LSMS) training and were intimately familiar with LSMS-style surveys. In some of the countries, enumerators furthermore had previous experience conducting surveys over the phone. To prepare enumerators specifically for conducting the HFPS, all interviewers received three days of standardized training ahead of the first round of the survey. Additionally, interviewers received a one-day follow-up training in between survey rounds. Regular audio audits ensured consistently high quality between interviewers. Interviewers used Survey Solutions' computer-assisted telephone interviewing (CATI) capabilities when recording the data.
Timing	• Burkina Faso: 5 Apr 2022 - 15 May 2022<br>• Kenya: 15 Nov 21 - 31 Mar 22<br>• Malawi: 03 Feb 2022 - 20 Feb 2022<br>• Nigeria: 29 Nov 2021 - 14 Feb 2022<br>• Tanzania: 30 Nov 2021 - 23 Dec 2021<br>• Uganda: 05 Aug 2022 - 29 Aug 2022
Data exclusions	No data were excluded from the analysis.
Non-participation	Non-participation rates are summarized in Table 1.
Randomization	Our study is observational and experimental randomization does not apply. Randomization in the selection of the sample is discussed above.

Reporting for specific materials, systems and methods

We require information from authors about some types of materials, experimental systems and methods used in many studies. Here, indicate whether each material, system or method listed is relevant to your study. If you are not sure if a list item applies to your research, read the appropriate section before selecting a response.

Materials & experimental systems

n/a	Involved in the study
☒	☐ Antibodies
☒	☐ Eukaryotic cell lines
☒	☐ Palaeontology and archaeology
☒	☐ Animals and other organisms
☒	☐ Clinical data
☒	☐ Dual use research of concern

Methods

n/a	Involved in the study
☒	☐ ChIP-seq
☒	☐ Flow cytometry
☒	☐ MRI-based neuroimaging
